# Case of a Pulmonary Polypus

**Published:** 1802-09-01

**Authors:** Eric Acharius


### *Case of a Pulmonary Polypus; by Dr.* ERIC ACHARIUS.

AFTER having premifed fome general remarks on Polypous Concretions, their origin, difference, and feat, the author communicates the following cafe:

S. B. A poor girl, aged 15 years, had been attacked about fix years before with violent vomiting, which increafed to fuch a degree, that fhe had no reft day or night, and as foon as fhe ate or drank any thing, the vomiting came on, attended with a griping and a painful fenfation at the heart, ceafing frequently at the approach of another paroxyfm of vomiting, which often recurred even when fhe had not taken any nourifhment at all. With the vomiting fhe ejected much mucus and bilious matter. Her ftrength decreafed confiderably, and fhe was obliged to keep her bed nearly fix months, without experiencing much relief from the remedies fhe was ordered to take. Afterwards, however, when fhe had almoft ceafed ufing any medicine, fhe got better by degrees; her appetite was reftored, and the ftomach could keep the ingefta without farther complaint. A year and a half fhe continued perfectly free from any complaint; after which time, however, fhe was feized with a periodical head-ach, and fome painful fenfations in the ftomach, without their being attended with vomiting; thefe complaints had ceafed, when fhe was attacked with an intermittent fever, which continued obftinate againft all remedies. Her ftrength and flefh wafted confiderably, particularly as a very frequent and troublefome cough fupervened, which feemed to fruftrate every medicine; but, in proportion as the cough increafed, the paroxyfms decreafed. Before the cough came on fhe felt herfelf much oppreffed, and her breath fhort and difficult. Some time after fhe brought up with the cough a polypus, without either blood or mucus, which almoft inftantly gave her great relief. Towards autumn the ague ceafed, and a little after the cough; fhe became quite well, and could leave her bed. Above fix months after, the cough returned, and the patient brought up with it a great number of polypous concretions: the cough was dry, and attended with a tickling fenfation in the throat, which always preceded the coughing up of the polypi: the cough returned from time to time. From this period, however, fhe feemed to make a rapid progrefs towards health. She is of low ftature, but lufty, and has by no means a fickly appearance. It is expected, that fhe will become much better with the appearance of the fluxus menfium. The colour of the polypi which this patient coughed up is white; their trunks are cylindrical, and fometimes a little compreffed, the extremities of which are broken broken and ragged, or divided into many ramifications; they are the fize of a common quill, fome lefs, and others bigger. The ramifications feemed exactly to correfpond with the bronchial twigs, which they moft probably fill up entirely; they are covered with a peculiar ftrong but thin membrane; when cut through they appeared to be compofed of fibres, placed thickly along each other, and their whole compofition feemed to be folid; when cut tranfverfely, they had not the appearance of being compofed of laminæ. They do not diffolve in water, until it becomes putrid, at which time they are macerated into a white mucus. No fimilar cafe is recorded by any author, except Dr. Warren, in Med. Tranf. Vol. i. p. 401.

